# Sasang Constitutional Medicine and Traditional Chinese Medicine: A Comparative Overview

**DOI:** 10.1155/2012/980807

**Published:** 2011-09-19

**Authors:** Junghee Yoo, Euiju Lee, Chungmi Kim, Junhee Lee, Lao Lixing

**Affiliations:** ^1^Department of Sasang Constitutional Medicine, College of Korean Medicine, Kyung Hee University, 1 Hoegi-Dong, Dongdaemun-Gu, Seoul 130-702, Republic of Korea; ^2^Family Medicine, Center for Integrative Medicine, University of Maryland School of Medicine, Baltimore, MD 21207, USA

## Abstract

Sasang constitutional medicine (SCM) is a holistic typological constitution medicine which balances psychological, social, and physical aspects of an individual to achieve wellness and increase longevity. SCM has the qualities of preventative medicine, as it emphasizes daily health management based on constitutionally differentiated regimens and self-cultivation of the mind and body. This review's goal is to establish a fundamental understanding of SCM and to provide a foundation for further study. It compares the similarities and differences of philosophical origins, perspectives on the mind (heart), typological systems, pathology, and therapeutics between SCM and traditional Chinese medicine (TCM). TCM is based on the Taoist view of the universe and humanity. The health and longevity of an individual depends on a harmonious relationship with the universe. On the other hand, SCM is based on the Confucian view of the universe and humanity. SCM focuses on the influence of human affairs on the psyche, physiology, and pathology.

## 1. Introduction

Interest in the use of complementary and alternative medicines (CAM) as alternatives to conventional Western treatments is increasing worldwide. The 2002 WHO West Pacific Region survey reported that 90%, 69%, 49%, and 45% of individuals in China, Korea, Japan, and Singapore, respectively, used traditional methods in personal medical care [1]. In the USA, an individual's CAM usage level including spiritual therapies increased from 33.8% in 1990 to 42.1% in 1997 and to 62.1% in 2002 [2]. In Europe, CAM treatment is common protocol for cancer patients, with over a third of the entire population reported using CAM methods, including herbal medication, homeopathy, and vitamin and mineral supplements [3]. In Europe, as in Asia, the range of CAM utilization varies from country to country, whereas approximately 15% of the population of Greece utilizes CAM, nearly 75% of the population of Italy uses CAM [3].

Of medical traditions categorized as CAM, traditional chinese Medicine (TCM) is prominently recognized. Yet, TCM is only one of numerous CAM modalities being utilized worldwide. Other significant medical traditions such as Sasang constitutional medicine (SCM) from Korea, Ayurveda from India, traditional Mongolian medicine, and traditional Vietnamese medicine are noteworthy. Sasang constitutional medicine is a unique system of medical constitutional typology that was developed towards the end of the 19th century by the Korean physician Lee Jema (1837–1900), whose life work is concisely summarized in two manuscripts, “*Dongeuisoosebowon (Longevity and Life Preservation in Eastern Medicine)*” and “*Gyeokchigo (Manuscript on Science)*.” In these works, Lee Jema delineates a medical constitutional typology which identifies an individual as one of the following four constitutional types: a Taeyang person, a Taeeum person, a Soyang person, or a Soeum person [61]. Contemporary applications for the field's rich tradition are being expanded extensively through research and development. Researchers are studying methods of diagnosis [62], conducting randomized clinical trials of constitutional acupuncture [63], and exploring approaches integrating western genetics into the eastern SCM framework [12].

Generally, over the course of world medical history, both eastern and western medical practitioners have been aware of or have used constitutional categorization as a tool to understand disease. Today practitioners prefer a standardized diagnostic approach which by principle assumes all individuals identical. Yet, awareness of unique constitutional characteristics is conducive to the prevention of disease, increasing effectiveness of treatment as well as decreasing adverse reactions to pharmaceuticals. For example, each individual's constitutional make-up determines weaknesses and strengths with respect to a hypoactive or hyperactive internal organ scheme, pathological developments, and an individual's reactions to herbal medicines. Disease prevention and health preservation regimens, therapeutic principles, and medicinal protocol are modified and personalized according to the individual's constitutional characteristics to provide the best health management. A well-developed system of constitutional typology will have medical benefits [62].

## 2. Methods

### 2.1. Search Strategy and Data Sources

The objective is to present a comprehensive introductory understanding of SCM. For this purpose, we reviewed the difference between TCM and SCM comparing medical philosophy, perspectives on the mind (heart), typological systems, pathology, and therapeutics of TCM and SCM. The following electronic databases were searched: Pubmed (http://www.pubmed.org /), DBPIA (http://www.dbpia.com /), Research Information Service System (RISS, http://www.riss4u.net /), and Korean Studies Information Service System (KISS, http://kiss.kstudy.com/). Search terms were Korean and English words relevant to Sasang typology such as Sasang typology, constitution, and Sasang medicine. In addition, we manually searched five Korean traditional oriental medical journals (Journal of Sasang Constitutional Medicine, Journal of Oriental Medicine, Korean Journal of Oriental Physiology & Pathology, The Korean Society of Oriental Medical Classics, and The Journal of Korean Oriental Internal Medicine (searched from their first publication date through December 2009)). To ensure accuracy, TCM and SCM primary source classical literature was also searched.

### 2.2. Article Selection and Data Extraction

#### 2.2.1. Article Selection

Peer-reviewed research articles on review papers and literature pertaining to medical philosophy, perspectives on the mind (heart), typological systems, pathology, and therapeutics were included. Articles that provided only clinical analysis, basic science, clinical case studies, translated text(s) or commentary on the history of SCM and Lee Jema, and constitutional symptoms or pharmacology of prescriptions were excluded.

#### 2.2.2. Data Extraction

All articles were reviewed by two independent reviewers (Hyensu Jang and Jiwon Lee), and data from the articles were extracted according to the predefined criteria. Information pertaining to medical philosophy, perspective on the mind (heart), typological system, pathology, and therapeutics was collected.

## 3. Results

**Figure 1:**
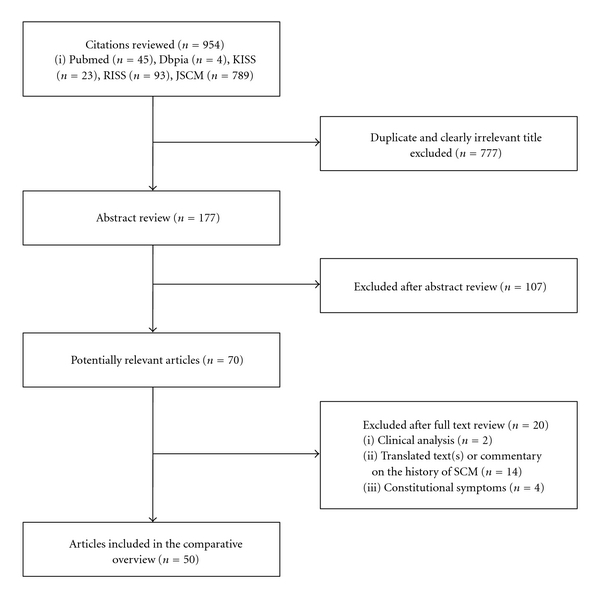
Flow diagram showing the number of studies included and excluded from the comparative review.

The search identified 954 potentially relevant articles. Seven hundred seventy-seven studies which were clearly irrelevant titles or duplicates were excluded. The majority of the excluded studies did not incorporate theoretical Sasang typology in their research question. One hundred seventy-seven remaining studies' abstracts were reviewed. Of those studies, 107 were excluded according to the exclusion criteria. Hard copies of 70 articles were obtained and read in full. After a full text review, a total of 50 studies were included in the current review (Figure 1). 

### 3.1. Philosophy: Nature-Centered TCM Based on the Yin-Yang Theory and Five-Phase Theory, and Human-Centered SCM Based on the Sasang Theory

Complex scientific and philosophical concepts are the foundation for many medical traditions. TCM is rooted in the Taoist view of nature and humanity introduced in the ancient Chinese manuscript, *Huangdi's Internal Classic* [6, 7]. The Yin-Yang theory and Five-Phase theory [8, 30] are fundamental principles of Taoism and are the theories from which TCM developed. SCM's development was influenced by most of the same philosophical theories as TCM. Yet, SCM can be distinguished as a separate medical system because SCM emphasizes and innovates upon certain principles and aspects of shared East Asian philosophies. 

TCM is based on central philosophical concepts. First, it perceives *qi* as the basic substance of the universe [15, 16]. Second, TCM is focused on the physical universe and its nature rather than on humanity or human nature [8]. Third, it states that the nature of the universe changes constantly. These changes are governed by the Yin-Yang theory and the Five-Phase theory; therefore, changes can be observed and predicted [16]. Fourth, an individual's health and longevity is determined by their conformity to nature [29]. In other words, the Taoist idea of *wu wei* (letting it be) is central to TCM treatment principles and preventive medicine (Table 1) [30].

SCM, first introduced in *Dongeuisoosebowon*, is fundamentally based on the Neo-Confucian views of nature and humanity. The theoretical rationale supporting SCM is derived from Sasang philosophy [4, 5, 9–11, 13]. Sasang philosophy adheres to the following concepts. First, SCM emphasizes the importance of humans and humanity over the importance of nature [8, 19]. Humans exist independent from nature and are influenced by two factors, their social environment and human affairs, which comprise of matters concerning self and others [8, 29]. Second, SCM is fundamentally grounded on the Sasang philosophy, which perceives that all phenomenon and all matter of the universe including humans can be classified on a four-axis schema (Sasang) of which one of the axis is activity, mind, body, or matter (AMBM) [20, 21]. Sasang theory initially separates human beings into two aspects: the mind and the body. The mind is subdivided into four concepts. The mind aspect focuses on sorrow, anger, joy, and pleasure, which as singular concept is defined as the Seong-Jeong (Innate Nature-emotional disposition). The body is subdivided into four regions. The body is divided into the lung, spleen, liver, and kidney systems. This SCM organ scheme theory forms the outline for human physiology based on this four-point scheme of the Sasang theory [22–26, 28]. Third, SCM takes individual variability into account. The mental and physical characteristics of an individual can manifest on the broadest level in four different ways. Highlighting physical characteristics as an example, the human body has a predetermined congenital four-organ systems that control the functional balance of physiology and pathology [8, 15, 17]. Fourth, SCM strives to preserve health and longevity through constitutional management. For example, SCM advocates lifestyle and behavioral changes. Thus, SCM extends beyond simple medical methodology. It emphasizes the importance of self-cultivation, health preservation, and disease prevention, the key to which can be found in lifestyles compatible with a given constitutional makeup [19, 29, 31, 32].

### 3.2. Mind (Heart): The Heart as One of the Five Viscera in TCM and the Heart as the Mental Body Controlling the Physical Body in SCM

In TCM, the heart is one of the five zang viscera representing the fire element. According to TCM theory, the heart is positioned in the thorax and connected to the small intestine via the meridian and collateral in what is termed the internal-external relationship. The term *heart* is used to designate the general outward manifestation of internal vital activities in the broader sense or the human consciousness associated with spiritual activity and cogitative processes in the narrower sense [33]. The concept of the heart carries a different multifaceted connotation in SCM. 

First, the heart (mind) governs the entire body, as the mental aspect that controls the physical aspect [19, 20, 34]. The heart (mind) functionally supervises the body by coordinating and controlling the body's physical system composition and function [19]. Furthermore, from the SCM perspective, the heart (mind) embodies many different associations. The Taiji-associated mind can be understood as the overall fundamental force that controls all things [13, 35–38, 41]. Through the Yin-Yang association, the mind can be understood as the mental aspect as opposed to the physical aspect. For example, the mental aspect can refer to the virtuous mind defined as the quaternary set of values: benevolence, righteousness, propriety, and wisdom; as opposed to the physical aspect such as selfish desires exemplified by a quaternary set of characteristics: crudeness, superficiality, greed, and indolence. Finally, in the Sasang association, the mind represents the mind axis of the AMBM scheme in the Sasang philosophy (Table 2) [20].

Secondly, while physical functions are carried out through the four physical organs of the lungs, spleen, liver, and kidneys, the mental functions manifest through the four mental components of sorrow, anger, joy, and pleasure (the Seong-Jeong). Lee Jema compartmentalized the mind into Seong (Innate Nature) and Jeong (emotional disposition), each of which manifests in four different forms, namely, sorrow, anger, joy, and pleasure [17, 19, 35].

Thirdly, variations in Seong-Jeong arise due to the infinitely unique combinations and permutations of human affairs and interactions. An individual's goal is to remain balanced through all circumstances internally and externally. Excessive deviation will exert physical damage on the body as a major contributing cause of pathogenesis [19, 42, 43].

### 3.3. Typology: Pattern Typing in TCM and Constitutional Typing in SCM

Constitution can be defined as “the aggregate of physical attributes and functional characteristics that is comprehensively forged by hereditary determinants and environmental factors” [17, 61]. Though the definition of constitution can vary from person to person, constitutional typing itself had been met with great interest and attention in medical circles, especially in TCM and SCM. Zhang Zhongjing (150–219), a renowned TCM physician, had categorized individuals into Yang-organ types and Yin viscous types. Kuang Tiaoyuan (since 1931 present), another TCM physician, developed a typological system that placed individuals into one of six somatotype categories (normal, dark and stringent, congestive and stagnant, dry and ruddy, slow and cold, and languid and shiny) depending on prominent clinical features [44]. Typology has been used as a tool not only in the past, but currently in fields related to human behavior such as psychology and anthropology.

A TCM typology system was first introduced in *Huangdi's Internal Classics*, which was presumably compiled in the 3rd century BC. Its second book in the two-volume collection, *Lingshu (Spiritual Pivot)*, contains material relevant to a pattern typology, including the theory of the Five Body Types (based on the Yin-Yang theory) and the theory of the Twenty-five Body Types (based on the Five-Phase theory) [30]. *Lingshu* also mentions morphological attributes of the five viscera and the six bowels in reference to physiology and pathology. Therefore, TCM typology is essentially a typological pattern system that groups similar symptoms into specific pattern types (Table 3). 

In contrast to TCM, SCM has a philosophical basis that explains the composition and movements of the universe through the AMBM schema and interprets the natural, social, and physical phenomena from a human-centered perspective. When applied to medicine, this Sasang philosophy compartmentalizes humans into two aspects—the mind (heart) and the body (lung, spleen, liver, and kidney). The functional strength and weakness of the organ systems are the physical aspect, and the natural state of the mind that manifests as four constitutional variations is the mental aspect. In other words, the physiology and pathology of the organ systems (lung, spleen, liver, and kidney systems) and the core principle Seong-Jeong (sorrow, anger, joy, and pleasure) are congenitally influenced differently for different constitutional types. Therefore, four constitutional types, Taeyang person, Soyang person, Taeeum person, and Soeum person, in SCM stem from variations of the aforementioned four physical and four mental differentiations. Indeed, SCM explains the constitutionally unique attributes (including physiology and pathology) and uses the same rationale to apply differentiated therapeutics to different constitutional types (Table 4) [13, 17].

### 3.4. Pathology: TCM Pathology Based on the Meridian and Collateral Theory and the Visceral Manifestation Theory, and SCM Pathology Based on the Fourfold Energizer Center Theory and the Four-Energy-Center Theory

In TCM, the causes of diseases are generally identified as external etiology or internal etiology. The theoretical basis for TCM pathology can be summarized by the meridian and collateral meridian theory, which is based on the twelve meridians and collaterals, and the visceral manifestation theory, which is based on the internal and external organs [30, 39]. TCM pathology has certain features that distinguish it from SCM pathology. TCM and SCM have different perspectives concerning the internal or external nature of the disease at the site of pathogenesis. The first feature is that pathological conditions occurring in the meridian and collateral meridian are generally understood to be external, whereas those occurring in the organ systems are considered to be internal [33]. Second, when describing the characteristics of the pathological condition, diseases of the meridian and collaterals are referred to in terms of heat or cold-related pathology, and diseases of the viscera and bowels are defined as excessive or deficient [30, 51]. Third, the diagnosis is dependent on the eight principles, namely, the exterior, interior, yin, yang, cold, heat, excess, and deficiency determinants. The eight principles are used as tools for the four examinations process. Both traditional Chinese and Korean diagnosis begins with the four inspections: observing, listening, smelling/tasting, and palpating/pulse taking. Finally, through inductive analysis of the data, the practitioner determines the pathological condition using the eight principles as diagnostic terminology [14, 33, 55]. 

SCM maintains a different outlook on the cause of illness. TCM emphasizes its five-organ theory while SCM emphasizes its four-organ theory [13, 17]. According to SCM pathogenesis, illnesses arise from a disruption of balance between the larger hyperactive and smaller hypoactive organ systems, schematically speaking. Lee Jema explained that Sasang pathological syndromes are caused by variations in the qi that arise from the inherent differences in the sizes of the internal organs and the imbalance of the rising and descending qi of the Seong-Jeong. Furthermore, in SCM, the mind aspect must be taken into account when examining pathogenesis. An emotional disruption of the Seong-Jeong physiological impacts the fourfold energizer centers, thus disrupting the proper larger hyperactive and smaller hypoactive four-organ-system scheme of the fourfold energizer centers resulting in pathogenesis [11, 39, 40, 46–50]. Second, the external-internal properties are determined by frontal/dorsal, upper/lower, and inner/outer qualities. The internal organs are assigned to the dorsal side, and pathological conditions associated with this site are recognized as external. Conversely, the external organs are assigned to the frontal side and pathological conditions associated with this site are recognized as internal [34, 40, 51]. Third, physical metabolism is bifurcated into Qi-Humor metabolism and Water-Food metabolism. The lungs and liver are associated with Qi-Humor metabolism, with the lung being the dispersive force of the body responsible for expiration, and the liver the convergent force of the body responsible for inspiration. The spleen and kidneys are involved in the Water-Food metabolism, with the spleen being the ascending body and the kidneys being the descending body. Therefore, given the corresponding organs, Qi-Humor metabolism pathology is common in a Taeyang person or a Taeeum person, whose organ functions rely on the balance between the lungs and the liver system. Water-Food metabolism pathology is common in a Soyang person or a Soeum person, whose organ functions rely on the balance between the spleen and the kidneys [19, 45, 46]. Fourth, SCM eight principles are exterior/interior, yin/yang, ascending/descending, and dispersive/convergent determinants. In SCM, a patient is scanned for signs and symptoms that suggest disharmonies in the ascent/descent or dispersion/convergence processes of the yin or yang energies in the external or internal systems (Table 5) [15, 17, 52].

### 3.5. Therapeutics: Fortifying Healthy Qi and Eliminating Pathologic Qi in TCM, and Regulating the Mind to Control Disease in SCM

Since approaches and perspectives to pathology differ, TCM and SCM use different treatment methods and therapeutic principles. For example, SCM therapeutics is not centered on fortification or reductions, which are important concepts in TCM. TCM explanations of pathology revolve around the eight principles: exterior/interior, yin/yang, cold/heat, and deficiency/excess. In TCM, excess and deficiency factor is most significant. Deficient healthy qi or excessive pathogenic qi can both cause pathogenesis. Following the same rationale, therapeutic methods most often take the form of fortification of a deficient condition or reduction of an excess condition [30].

However, according to SCM, the primary cause of disease lies in an innate imbalance between the functional strengths of the organ structures, the default schematic setting of which is congenitally different among different constitutional types [8, 53]. The goal of therapy is to restore the original constitutional balance [34, 56, 57]. After the energy levels of the organ systems are balanced, the deficiency or excess are easily regulated, which is why SCM does not necessarily stress fortification or reduction in its herb prescription and therapeutic techniques. SCM combines all disease symptoms and indicators into a comprehensive set within the framework of a constitution to manage an individual's health, to develop the basis for treatment, and to prevent disease.

Imbalances in both the body and mind are another primary factor that instigates the loss of health. SCM does not limit therapy to externally caused diseases or rely solely on herbal medication as its therapeutic method. Compared to other CAM modalities, it emphasizes the therapeutic significance of psychological factors; therefore, its techniques are designed to control and contain joy, anger, sorrow, pleasure, love, hate, and desire. In other words, the mind is controlled to regulate the illness manifesting symptomatically in the body [12, 17, 58, 59]. Regulation is possible through interaction with others and rectification of self, which enables the mind to regulate illness [18].

Although it is important to regulate one's self since this is not always possible, Lee Jema also developed intricate prescriptions to help each constitution with naturally weak areas prone to imbalance and disease. When applying acupuncture and herbal prescriptions, the primary therapeutic goal is to balance one's healthy energy. Therefore, SCM herbs are used for regulating and balancing the healthy qi of the body whereas TCM methods emphasize tonifying and sedating according to strengths of the pathogenic qi and the healthy qi [8, 27].

Furthermore, Lee Jema provided several examples of the limitations of TCM therapeutics, especially when addressing Taeeum persons and Taeyang persons pathologies [19]. While treating patients of the Soyang type and Soeum type, Lee Jema found that many TCM approaches effectively addressed concerns. However, TCM therapeutics and herb prescriptions delivered minimal results for the Taeeum person and Taeyang person types. Lee Jema created new therapeutics and prescriptions for individuals of this constitutional type.

Finally, SCM shares the same vision as tailored medicine. Individuals are cared for with individualized treatment plans, which takes into complete account their unique health factors. SCM also promotes individualized self-regulation as a preventative measure for that individual's specific susceptible high risk chronic diseases. For example, the natural limitations of an individual due to the hypoactive “small” visceral organ should regulate given situation through specific constitutional medication, diet, physical training, and psychological caution (Table 6) [13, 60, 64].

## 4. Discussion and Conclusions

TCM and SCM are medical traditions originating from oriental philosophical perspectives. TCM is based on the Taoist views of the universe and humanity introduced in *Huangdi's Internal Classic*. Briefly, qi are the basic substance of the universe, and the generation, degeneration, variation, and operation of qi is governed by the Law of Yin-Yang and the Five-Phase theory. Thus, the healthiness and longevity of an individual, a microcosm of universe, depends on a harmonious relationship with the universe, the macrocosm. SCM is based on the Neo-Confucian views of the universe and humanity introduced in *Dongeuisoosebowon*. Neo-Confucianism also played an important role in the development of TCM as well. The work of the Yuan dynasty physician Zhu Danxi (1280–1358) is often cited as a typical example of Neo-Confucianism influencing TCM. His medical writings state that emotions are a key factor in the causation of illness, Yet, he did not further develop a pathophysiology and typological system from his understanding of emotions as a key factor in the causation of illness [13, 18].

The fundamental concepts of SCM include both emotional and physical aspects. The emotional and physical factors are integrated to the extent that the concept of “heart” and “mind” are viewed as nearly being synonymous. The concept of the mind (heart) is distinctly different in SCM and TCM. In TCM, the heart is one of the internal organs representing the fire element. In SCM, the mind (heart) governs the entire body, which suggests that the mental component controls the physical component. The mind is compartmentalized into the Seong and the Jeong that manifests in four forms, sorrow, anger, joy, and pleasure. In SCM, the primary cause of illness is disruptions to the Seong-Jeong (Innate Nature and emotional disposition) of sorrow, anger, joy, and pleasure that arise from human interactions and self-control. SCM emphasizes preservation of health through daily health management based on constitutionally differentiated regimens. Additionally, SCM has qualities of preventive medicine through emphasis of patient-centered self-cultivation of the mind and body.

In TCM, individual unique characteristics are explained by a pattern typology, which is categorized by somatotype, disposition, symptomology, and pathology according to outward similarities. Conversely, in SCM, individual unique characteristics are explained by a systemic typology that combines somatotype, disposition, symptomology, physiology, pathology, therapeutics, and prevention into a comprehensive picture based on the constitutional differentiations of each individual. For this reason, the TCM typology is a pattern typology, whereas the SCM typology is defined as a constitutional typology. Overall, SCM is a well-developed and comprehensive system of constitutional typology that explains the distinct constitutional qualities of each constitutional type, including the physical form, mental disposition, physiology, pathology, symptomology, diagnostics, therapeutics, and preservative methods, with a consistent rationale.

TCM established the “Differentiation of the Syndrome Theory” and analyzed each symptom, classifying it into symptomatic types. If patients with the similar dominant complaint showed different symptomatic types, they were treated with different therapies. Although traditional Korean medicine was influenced by TCM in many aspects, it progressively developed a unique constitutional view that mind and body are inseparable and the physical state of the human body can be remarkably changed by mental factors. This belief of traditional Korean medicine led to SCM in the latter half of the nineteenth century [34].

TCM pathology is based on the meridian and collateral meridian theory, which is related to the twelve meridians and fifteen collaterals, and the visceral manifestation theory, which is related to the five viscera and the six bowels. Diagnostics begin with the four examinations, after which clinical data is inductively analyzed before proceeding to the eight principles. The eight principles is then used to determine the therapeutic principle and method. The SCM pathology is based on disruptions of the fourfold energizer centers arising from deviations of Seong-Jeong. The fourfold energizer centers house the four organ systems, which each has a correspondence system including the internal and external organs carrying out vital metabolic processes. In the SCM, there are two metabolic systems, the Qi-Humor metabolism, which is controlled by the lungs and the liver, and the Water-Food metabolism, which is controlled by the spleen and the kidneys.

TCM therapeutics is based on fortification of the healthy qi and elimination of the pathological qi. On the other hand, the SCM therapeutics is designed to regulate the mind to control the illness. In SCM, interacting with others and rectifying oneself allows the Seong-Jeong of sorrow, anger, joy, and pleasure to be controlled and contained through self-cultivation, which enables regulation of the mind to regulate the body. 

When the current study reviewed the differences between the TCM and the SCM, it included all peer-reviewed articles and included only information relevant to the objectives of the study. Future reviews should be conducted to systematically organize SCM beyond an introductory level. In addition, we could not provide enough solid peer-reviewed references for the claims in SCM not to mention the TCM. We hope that interest in SCM stimulates more studies addressing areas that have not been peer reviewed.

## Figures and Tables

**Table 1:** TCM and SCM philosophical foundations.

	Traditional Chinese medicines (TCM)	Sasang constitutional medicine (SCM)
		*Neo-Confucian Views*
Background	*Taoist Views * Choi et al. [6]: The foundation of most traditional eastern Asian philosophies and sciences originated from the concepts introduced in *I Ching (The Book of Changes) * Song [7]: TCM adapted and expanded upon the Taoist philosophy of the universe reconceptualizing how the macrocosmic universe manifests within the microcosmic sphere of human physiology Chae et al. [8]: Traditional Chinese Medicine is based on Taoism and explains the universe through the Yin-Yang theory and the Five-Phase theory	Kim et al. [4]: Lee Jema, the founder of Sasang philosophy, was a follower of the Realist School of Neo-Confucianism and was influenced by his teacher Dasan Jung Yakyong (1762–1836). Sasang philosophy initially draws upon ideas about the human body from the *Internal Classics Lingshu* including the theory of Yin and Yang and the theory of the twenty-five body types. The philosophy incorporated ideas concerning the mind from Mencius, who taught about four vices: ignobility, frivolity, avarice, and timidity Song [5]: According to Sasang philosophy, one strives “to value good and to scorn evil,” and “to understand others and to rectify oneself,” through a righteous steadfast mind to be in control of one's mind. This philosophy is rooted in Mencius's concepts of the “Unmoving Mind” and “Collecting the Mind.” As a result, the philosophy of “Rectification of the Self through Controlling the Mind,” led to the Sasang therapeutic approach of “Controlling the Mind to Heal the Body”. Therefore, according to Sasang philosophy, one will be able to control one's illness and rectify oneself when one returns to and maintains the state of a righteous mind Song [7]: The main philosophical basis of Sasang philosophy is human nature and interpersonal relationships rooted in Confucian foundations within a completely human-centered paradigm Park and Song [9]: Korean Sasang medicine is based on Neo-Confucian philosophy and the *Dongeuibogam (Treasured Mirror of Eastern Medicine)*, a Korean medical text Yeo [10]: Sasang typology, which describes nature through a quaternary system, is based on a combination of Neo-Confucianism and the medical traditions of Korea Song [11]: The TCM constitutional approach was first mentioned in Chapter 72 of *LingShu* of the *Inner Classics* as five body types. In 1894, Lee Jema presented SCM, which adapted or refuted ideas from the classics. For example, Lee Jema stated among the five body types, Greater Yang type, Lesser Yang type, Greater Yin type, Lesser Yin type, and Yin-Yang balanced type, described in the *Inner Classics*, that the Yin-Yang balanced type, a perfect human type, did not exist. Moreover, he claimed that this classification was impractical from a clinical viewpoint Kim et al. [12], Kim and Pham [13], and Kim et al. [14]: The mind-body interrelationship reflects deeply ingrained Neo-Confucian ideologies such as its concepts of morality, the Four Virtues: Benevolence, Rightness, Propriety, and Wisdom

Assumption	*Qi as the Basic Substance of the Universe * Lee [15]: Based on the macro- and microcosmology, the human being is defined as a microcosm within the macrocosm, and this concept overrides the specificity and individuality of each human being Giovanni [16]: Qi is present in all objects and provides the metaphysical and physical material that comprises humans. Therefore, regardless of its extrinsic form, the intrinsic qi is universal and the same	*Individual Variability * Lee [15]: Sasang philosophy describes the human being as a progressive manifestation of the four constitutional differentiations of the lesser and greater organ structure scheme that is formed through the expression of the innate Seong-Jeong (Innate Nature and Emotional Disposition) of Sorrow, Anger, Joy, and Pleasure Chae et al. [8]: It also explains individual differences in patterns of behavioral tendencies and physical characteristics based on particular biological and psychological traits Song et al. [17]: The external causes of illness (wind, cold, summer heat, dampness, dryness, and heat), the internal causes of illness (food, fatigue, and other miscellaneous causes), and the mental influences(sorrow, anger, joy, and pleasure) do not affect all individuals to the same degree. Different individuals with different constitutional makeups exhibit varying levels of vulnerability and responsiveness *Classification of Individuals * Leem and Park [18]: (SCM) classifies people into four constitutional types primarily based on an individual's psychological and physical traits. Susceptibility to diseases and response to drugs vary upon (an) individual's constitution. The constitutional type is very much the same as the genetic polymorphism which is determined at birth and remained unchanged throughout one's life

	*Nature*	*Humans*
Focus	Chae et al. [8]: Traditional Chinese Medicine places importance on harmony between humanity and nature Giovanni [16]: The universe originates from qi, and the formation, extinction, variation, and movement of qi are governed by a uniform law and order. Because the humans are microcosms originating from the same qi, they have the same integrity and identity as the macrocosm and are governed by the same laws as the universe	Choi [19]: Human matters influence the physiological and pathological conditions. In Dongeuisoosebowon (A Discourse on the Origin of Eastern Medicine), (Lee Jema) states, “Most doctors in the past did not understand that diseases arise from disruptions of the mind such as love, hate, desire, joy, anger, sadness and pleasure,” and that the Innate Nature (Seong) and the emotional disposition (Jeong) that arise from involvement in human affairs are the primary causes of illness Chae et al. [8]: Sasang typology emphasizes harmony in (one's) social life and developing one's character

		*Sasang Theory*
		Ji [20]: The TCM Pathway, Taiji → Yin-Yang → Sasang, is reinterpreted in the Sasang Philosophy as Mind → Mind-Body → Activity-Mind-Body-Matter Kim [21]: The Sasang (four principles) are derived from the I Ching, which describes the development of Taiji into Yin-Yang into Sasang
		*Perspective*
		Choi and Park [22]: The “theory of uniformity external factors and internal factor” states that the source of the externally manifested factor originates from an individual's internal mind, specifically the Seong-Jeong. In SCM morphology-based therapeutics, deviation of the Seong-Jeong is the foremost important factor in morphological expression
Main theory	*Law of the Yin-Yang and the Five Phase * Giovanni [16]: Changes in the human body can also be observed and predicted within the Yin-Yang and the Five-Phase theories Kim and Song [23]: In Huangdi's Internal Classics, the Five-Phase theory, external morphology and symptoms are classified according to their corresponding five visceral zang organs	Kim and Song [23]: According to SCM four-axis schema (Sasang) of Activity-Mind-Body-Matter theory (AMBM), external morphology and symptoms are classified by characteristics of the relative “largeness” and “smallness” of the internal organ schema, Seong-Jeong, and Disruptive Desires, or greed. The morphology in Sasang constitutional medicine can be said to have typological implications Song [24]: Lee Jema derived his system based on Confucian concepts of Seong-Myeong (pertaining to the human being as a predetermined and preordained existence, inherited congenital aspects) and Ji-Haeng (pertaining to the human beings as a self-governing and self-preserving existence). … he proposed a method of self-cultivation in speech and conduct, setting a novel code of conduct also suggested constitutionally differentiated ways in Ji-Haeng based on the constitutional differentiation according to Sasang constitutional medicine, proffering a new paradigm in medical therapeutics. When deviating from the ethical and moral lifestyle because of temptation, one can fall prey to disease. If one's thoughts and actions are improper and indolent, this can trigger pathological conditions Lim et al. [25]: The Activity-Mind-Body-Matter Sasang theory is the framework which explains Lee Jema's perception of the human being. The human being is initially divided into a Mind-Body dichotomy. The Seong (Innate Mind) reflects in the Mind and is associated with knowledge, and Hyeong (Expressed Form) is the expression of speech and conduct within and is associated with action. The internal self Seong is divided into mind and body: everything outside of self (not self); Hyeong is divided into activity and matter, forming an Activity-Mind-Body-Matter quaternary Lee [26] and Lee et al. [27]: Humans are defined as either self or not of self, literally “outside of self.” Self is further specified as Mind and Body; outside of self is Activity and Matter Kang and Park [28]: One elementary paradigm of Sasang theory is the duality of form and function. The Heaven/Human-Seong/Myeong grouping of the quaternary is found in the structural scheme of the Sasang quaternary, whereas the Heaven/Seong-Human/Myeong grouping of the quaternary is found in the functional scheme of the Sasang quaternary. This paradigm can be applied to interpreting social life as well as an individual. If the structural scheme forms the basic elements in life, the functional scheme is involved in making life distinctly human

Health and longevity	*Conformity to Nature * Jeong et al. [29]: The classical texts emphasize that the most important aspect of self-restraint is to be in control of one's mind. Such ideas are found in the Dongeuibogam and were practiced as part of the Taoist tradition Jeong et al. [29]: In the Taoist tradition, wu wei (letting it be) is essential to maintain self-restraint. The Dongeuibogam states that self-restraint is of utmost importance. The factors that influence self-restraint are age, original qi, congenital state of health before birth and environmental factors after birth. (preheavenly and postheavenly qi) Han and Li [30]: A pathological condition is essentially a state of disharmony with nature; therefore, the purpose of therapy is to restore harmony with nature	*Self-Cultivation * Choi [19]: Lee Jema explains that the penchant for indulgence in the Four Vices (drinking, sensual pleasure, money, and power) can be a life threatening. (Lee Jema) expounds that “the Four Virtues of attentiveness, simplicity, knowledge-ability, and vigilance leads to longevity,” because “he who values attentiveness can avoid indulgent drinking, he who values simplicity can avoid temptation for sensual pleasures, he who values knowledge-ability can avoid hoarding of wealth, and he who values vigilance can avoid striving for power” Jeong et al. [29]: One should act cautiously after contemplative introspection. Lee Jema believed the cardinal rule when interacting with others to behave in a manner which improves and harmonizes everyone's life Jeong et al. [29]: Life expectancy is greatly determined by lesser hypoactive organ's functional capacity. In addition to life expectancy, a person's the lesser organ's functional capacity determines the quality of health as well as severity of illness. A person is born with a defined amount of preheavenly qi. During childhood, adolescence, and early adulthood, one is able to sufficiently live with no qualms with postheavenly qi from food and stores of excess qi in the four visceral organs. After one's body weakens in middle life and old age, then, one is unable to maintain sufficient energy from postheavenly qi. Then one must draw upon previously stored excess qi to balance the difference. As one ages, he is unable to continuously or indefinitely draw upon or efficient use stored qi. Therefore, to regulate and increase the quality of qi, one must control his mind (*침심*). The better one is able to control their mind, the more efficient his use of qi and quality of life will be. This philosophy is based upon the idea that the mind and body are one unit and the mind can control the functions and quality of the body Yoo et al. [31]: Exercise self-restraint in order to extend one's lifespan. Cultivate self-restraint by controlling one's mind or understanding of others. A person's lifespan is a measure of how well that person cultivated self-restraint. Sorrow, anger, joy, and pleasure emerge from social relationships. If one is able to successfully navigate social relationships and use self-restraint, then one will be able to foster a longer lifespan. If one cannot successfully navigate relationships of society, then one will harm the qi of the four visceral organs. When this happens, one's life expectancy decreases. Therefore, to extend one's life expectancy, it is essential to preserve one's prone lesser organ through controlling one's mind and understanding others Song [32]: By avoiding harm to the lesser hypoactive organ and virtuously controlling one's mind and interactions with others to build postheavenly qi, one is able to compensate for the shortcomings of the lesser hypoactive organ and extend one's life expectancy. One's life expectancy can fluctuate based on adjustments to one's self-restraint. Therefore, based on a person's ability to manage himself, he will prevent potential weaknesses and illnesses. As a mind-centered approach committed to amplifying wellness of the mind, through treating the mind, a physician is able to indirectly treat the organs and extend the patient's life

**Table 2:** The Significance of the* heart* in TCM versus SCM.

	Traditional chinese medicines *(TCM) *	Sasang constitutional medicine (SCM)
Attribution	*One of the Five Viscera * Xinngon [33]: …“the heart rules the blood and blood vessels, stores the spirit, and opens into the tongue.”… “the heart's brilliance manifests in the face.” The statement that the heart rules the blood and blood vessels indicates that the heart is responsible for regulating the flow of blood within the vessels	*Mind * Choi [19]: …the mind is the controller of the body, positioned in the nook in the middle of the back, facing straight ahead toward the chest, shining its brilliant light. The ear-eye-nose-mouth has nothing it cannot observe, the lung-spleen-live-kidney has nothing it cannot comprehend, the chin-chest-navel-belly is all-attentive, and the head-arm-waist-leg is all-vigilantJi [20]: Lee Jema defines the general and specific concepts of the mind (heart) in one of his writings, *Gyeokchigo: Caution on Insincerity*, as follows: “Taiji is the mind, Yin-Yang is the mind-body, and Sasang is the Activity-Mind-Body-Matter.” This progressive differentiation of the mind in the Taiji-associated mind from the Yin-Yang-associated mind and the Sasang-associated mind is similar to the Taiji, Yin-yang, Sasang, and Eight Trigrams differentiation in the *I Ching *Shim et al. [34]: Lee Jema also emphasized that the human mind and body are not separate. They closely reflect each other. He mentioned in the “Origin of Oriental Medicine” of *Dongeuisoosebowon* that a disease can be provoked by environmental factors, but of more importance is the influence of psychogenic factors such as sorrow, anger, gladness, and enjoyment. Lee emphasizes the importance of regulation of the mind during treatment, especially in chronic diseases, assuming that immune system dysfunction and psychogenic disorders are the main causes of chronic disease

		*Seong (Innate Nature) and Jeong (Emotional Disposition)*
		Choi [19]: The Seong-Jeong of sorrow, anger, joy, and pleasure can damage the balance of internal organs and lead to pathologies. Lee Jema states, “If the Qi of sorrow and anger engage in unchecked movement, it will ascend improperly and gather in the upper sectors; if the Qi of joy and pleasure engage in unchecked movement, it will descend improperly and gather in the lower sectors. When the ascending Qi moves improperly to gather in the upper sectors, the liver and kidney are injured; when the descending Qi moves improperly to gather in the lower sectors, the spleen and lung are injured”
The mind	*Consciousness * Xinngon [33]: The human consciousness is associated with spiritual activity and cogitative processes (in the narrower sense).	Song et al. [17]: Seong is the component of the mind related to “the self,” while Jeong is the component of the mind related to that which is externalKang and Park [35]: Seong-Jeong is a fundamental theory of SCM physiology, pathology, and treatment theory. Seong-Jeong manifests as sorrow, anger, joy, and pleasure
		Kim [36]: The “mind” encapsulates two ideas: (1) the mind is the fundamental force that controls all things and (2) as the mind is the central core of the body, the mind is the center of the universe Son and Song [37]: … the body is controlled absolutely by the mind Lee and Song [38], Lee and Kim [39], and Lee and Song [40]: Fundamentally all things of the cosmos is Taiji, and at the core of its essence Taiji is the mind Hwang et al. [41]: Yin-Yang theory is one possible method to perceive man. The duality of man is defined as Innate Nature and emotional disposition. A man's Innate Nature and emotional disposition determine the lesser and greater organ which subsequently determines one of the four Sasang constitutional typesKang and Park [35]: From the Taiji perspective, man is defined essentially as mind. The mind is the essence of man as well as the physiological and anatomical heart of the body Kim et al. [12], Kim and Pham [13], and Kim et al. [14]: SCM regards the heart as the king among the five viscera, which is equivalent to the mind Kim [42]: Covetous desire is when the mind's desires exceeds that which the body needs. More specifically, covetous desire is when the mind and body deviates from what is proper virtuous “knowledge” and “behavior” Kang and Park [43]: To be virtuous, one must abstain and diminish personal desires and follow the righteous path

**Table 3:** Characteristics of TCM typologies.

	Traditional chinese medicines (TCM)
	*Fiv-Somatotype Typological System (Taeyang, Taeeum, Soyang, Soeum, or Equilibrated)*
Lingshu	Song et al. [17]: In the five-somatotype typological system, individuals are placed in one of the following five categories based on the internal Yin-Yang balance: Taeyang, Taeeum, Soyang, Soeum, or Equilibrated. The twenty-five-somatotype system concerns the predominant elemental-phase associated with an individual (Wood, Fire, Earth, Metal, and Water), and each of the five categories are further split into five subsets *Morphological attributes of the five viscera and the six bowels * Wang [44]: Lingshu also mentions the morphological attributes of the five viscera (large, small, high, low, firm, soft, upright, and tilted) and the six bowels (small, large, long, short, thick, thin, convoluted, straight, flaccid, and tight) in relevance with physiology and pathology

Zhang Zhongjing (150–219)	*Yin and Yang Viscous Types * Wang [44]: There are more individuals with the Yin viscous types, and such individuals are more prone to illnesses. Alternatively, individuals with the Yang viscous types, who are relatively scarce in number, are unlikely to become ill. Ironically, the Yin types tend to take better care of their health due to their congenital vulnerability to sickness and end up to be bed ridden less often, while the Yang types tend to take less care of their health due to their naturally good health and eventually fall prey to sickness

Kuang Tiaoyuan (since 1931 present)	*Six Somatotype Categories (Normal, Dark and Stringent, Congestive and Stagnant, Dry and Ruddy, Slow and Cold, and Languid and Shiny) * Wang [44]: The typical “normal” type would display sufficient strength in the limbs, a healthy complexion, good digestion, resistance to temperature change, slight dryness of the mouth, normal urination and defecation, a strong pulse, and normal tongue condition

**Table 4:** Characteristics and correspondences according to constitution.

	Taeyang person	Soyang person	Taeeum person	Soeum person
Hyperactive organ^a^	Lung	Pancreas	Liver	Kidney

Hypoactive organ	Liver	Kidney	Lung	Pancreas

Distribution	0.001%	30%	50%	20%

Character	Creative Progressive Charismatic Heroic	Moody Easily bored Sacrificing Righteous Hot tempered	Pragmatic Endurable Humorous Reflective Cautious	Neat, Mild Logical Organized Egocentric Persistent

Body shape	Developed nape of the neck, Slender waist	Developed chest, Small hip	Thick waist, Weak nape of the neck	Developed hip, Weak chest

Healthy sign	Normal urination	Normal bowel habit	Normal sweating	Normal digestion

Unhealthy sign	Bubbles in mouth, Emesis	Constipation	No perspiration	Indigestion

Nature	Sorrow	Anger	Joy	Pleasure

^a^From the SCM perspective, the lung, pancreas, liver, and kidney represent a functional system of organ groups that represent respiratory, digestive, preservative, and excretive systems, respectively. This table is modified from Chae et al.

**Table 5:** A Comparison of TCM and SCM pathologies.

	Traditional chinese medicines (TCM)	Sasang constitutional medicine (SCM)
Physiological principles	Zang and Fu Viscera	Interregulatory Relationship of Visceral Groups

	Han and Li [30]: TCM describes the human internal visceral system, which consists of two groups, zang and fu viscera, based on the concept of the five elements of the material world: fire, metal, wood, earth, and water. The zang viscera include the heart, lungs, liver, spleen, and kidneys: they are in a mutual relation to the corresponding fu organs, the small intestine, the large intestine, the gall bladder, the stomach, and the urinary bladder, respectively. By the rule of the five elements, the proper functioning of zang and fu viscera in an interactive circular principle maintains the balance between Yin and Yang in the human body, the essential condition for health Lee and Song [38], Lee and Kim [39], Lee and Song [40]: Cold symptoms and hot symptoms appear in all humans equally	Kim et al. [12], Kim and Pham [13], and Kim et al. [14]: … visceral groups are the lung, kidney, liver, and spleen groups. The lung group includes lungs, tongue, esophagus region, ears, brain, and skin. The spleen group consists of spleen, stomach, breasts, eyes, and tendon. The constituents of the liver group are the liver, small intestine, nose, lumbar region, and muscles. The kidney group has the kidney, large intestine, urethra, bladder, mouth, and bones. Among these groups, it is believed that specific interregulatory relations are present between specific pairs of visceral groups. As such, visceral groups form two pairs: one set consists of the spleen and the kidney pair and the other set is composed of the lung and the liver pair. The relationship between each pair can be compared to the unbalanced state of a seesaw. As one visceral organ has a natural hyperactive state, its counterpart has a comparatively deficient hypoactive state *Fourfold Energizer Centers * Song [7]: The fourfold energizer centers are the superior abdomen, upper mid, low mid, and the inferior abdomen. Physiologically phenomena of sorrow, anger, joy, and pleasure function according to the principle of Yin-Yang-ascension-dissension (ascent/descent) within the fourfold energizer centers internal system Choi [19]: *Dongeuisoosebowon *illustrates the positioning of the fourfold energizer centers within the comprehensive quaternary organ scheme … “the lung and esophagus comprise the upper-most burn-center, the spleen and stomach comprises the upper-middle burn-center, the liver and small intestine comprise the lower-middle burn-center, and the kidney and large intestine comprises the lower-most burn-center” *Water-Food metabolism * Chi and Ahn [45]: The ascension-dissension principle is when metabolized nutrients (Water-Food) create four energies (warm-hot-cool-cold) which ascend and descend in the body Cho [46]: The four Sasang zang organs the lung, spleen, liver, and kidney have specific attributes. All body functions such as inhalation/exhalation, or nutrient consumption and waste discharge abide according to the specific attributes of the four zang organs Choi [19]: Water-Food passes through the four external organs (esophagus, stomach, small intestine, and large intestine), where they are converted into the four energies (warm, hot, cool, and cold), which then disperse and travel to the conforming organ systems to be converted into basic vital material. For example, the warm energy of water and nutrients is converted into Jing material in the esophagus, after which it enters the tongue to form the Pool of Jing. The light component of the Pool of Jing then exits through the ear and becomes Shin material, which subsequently enters the cranium to form the Pool of Ni. The lighter juices of the Pool of Ni then fortify the roots of the lung, whereas the heavier dregs support the esophagus and form the skin

Theoretical basis	The Twelve Meridians and the Internal and External Organs	Inherent Differences in the Sizes of the Internal Organs and the Disruption of Seong-Jeong Choi and Kim [47]: The greater zang organ's physical qi and blood and functional operation is abundant and can easily become excessive. The lesser zang organ's physical qi and blood and functional operation is deficient

Site of pathogenesis	Xinngon [33]: The six excesses come in contact with the external energy of the individual through the meridian and collateral and trigger the external disease, whereas dysfunction of the organ systems, where foodstuffs are processed and assimilated into the system in the form of internal energy, are the cause of internal disease	Lee et al. [48]: The four zang organs are damaged by sorrow, anger, joy, and pleasure. The sorrow, anger, joy, and pleasure of heaven and earth is called Seong and develop into exterior disease. The sorrow, anger, joy, and pleasure of human affairs is called Jeong and develop into interior disease Lee and Song [38], Lee and Kim [39], and Lee and Song [40]: Seong is the root cause of exterior diseases and develops slowly becoming chronic diseases. Jeong is the root cause of interior diseases and develops rapidly becoming short-term diseases Kim and Song [49]: The healthy balance and maintenance of sorrow, anger, joy, and pleasure can increase the function of the zang organs. By increasing the function of the zang organs, the overall health of the individual increases Song [11]: The physiological and pathological features of each constitution are determined by hyperactive and hypoactive states of visceral groups that are believed to be intrinsic and congenital. The hypoactive part is more important. If this congenital shortcoming is controlled well, a healthy state can be accomplished Hwang and Koh [50]: Greed is the one of the root causes of disease, specifically greed with respect to human relationships. When a person is able to control their emotions and their emotions are in balance, then greed does not move the heart of the person. Greed develops through unbalanced relationships in the external environment, for example, human relationships. If a person's mind is unbalanced, then the external greed will become embedded into the mind. When this external greed settles into the mind, the Jeong is influenced and the four ruinous vices appear. The four ruinous vices are gluttony and alcoholism, lust, avarice, and excessive ambition. These four ruinous vices harm the Jeong. Symptomatically the four vices present in the body as internal diseases

Characteristics	*Diseases of Meridian *(*Hot or Cold*)* and Diseases of Organs *(*Excess or Deficient) * Han and Li [30]: Diseases of the meridian and collateral are referred to in terms of heat, or cold-related pathology, whereas diseases of the internal and external organs are defined as excessive or deficient *External-Internal Symptomatology * Kwak et al. [51]: In TCM, the root cause of disease and pathogenesis is categorized as internal or external. If it is an internal disease, then the disease is considered to be a chronic disease, and if it is an external disease, then the disease is considered to be a short-term (infectious) disease. Internal disease can progress and exhibit external symptoms, and external disease can progress and exhibit internal symptoms	*External-Internal Properties are Determined Frontal/Dorsal, Upper/Lower, and Inner/Outer Qualities * Song et al. [17]: The sublumbar region is governed by the kidney, and pathological development related to this locus is understood to be external in nature, while the subumbilical region is governed by the large intestine and pathological conditions related to this locus are understood to be internal in nature *Original Symptomatology *(*素證*) Lee and Song [38], Lee and Kim [39], and Lee and Song [40]: The original symptom can progress further into a disease. The original symptom characteristics will remain or increase. For example, if the original symptom was characterized as deficiency, then the disease will remain deficient in nature *External-Internal Symptomatology * Kwak et al. [51]: In SCM, internal and external diseases are the accumulation of multiple symptoms. Internal diseases will develop not only from internal diseases, but also from a combination of internal and external symptoms. Therefore, internal disease symptomology includes both internal and external symptoms. Similarly, external diseases develop from a progression of both external and internal symptoms

Diagnosis	*Eight Principles (exterior/interior, yin/yang, cold/heat, and excess/deficiency) * Giovanni [16]: The eight principles are the most basic diagnostic method that can be used to ascertain the site and properties of the pathology or the balance between healthy qi and pathogenic qi. In addition, this diagnosis is used to collect other relevant information needed to determine the therapeutic principle *Classification of Symptomatic Types * Xinngon [33]: Traditional Chinese medicine (TCM) established the “differentiation of the syndrome (*辨證*) theory” and analyzed each symptom, classifying them into symptomatic types (*證型*). If patients with similar dominant complaints showed different symptomatic types, they were treated with different therapies Kim et al. [12], Kim and Pham [13], and Kim et al. [14]: In TCM, symptoms are differentiated to diagnose disease into disease types and treated accordingly	*Eight Principles (Exterior/Interior, Yin/Yang, Ascending/Descending, and Dispersive/Convergent) * Kim and Song [52]: The pathological eight principles of SCM are exterior/interior, yin/yang, ascending/descending, and dispersive/convergent. The movement of exterior/interior, yin/yang is ascending/descending, dispersive/convergent Lee and Song [38], Lee and Kim [39], and Lee and Song [40]: The movement of exterior/interior, yin/yang is ascending/descending, dispersive/convergent. The eight principles of each constitution is specific to each particular constitution. Each eight principle can be classified as cold or hot, regardless of whether it is external or internal. For example, the Soeum type can have an external disease that is cold Song et al. [17]: The Soeum person external disease occurs when the external yang energy fails to ascend smoothly, and the Soeum person internal disease occurs when the internal yin energy fails to descend smoothly *Classification of Constitutional Types * Song et al. [17]: SCM classifies constitution according to the traits of an individual's mind and body, indicating that personal sensitivity to a certain drug can be different according to physical constitution

**Table 6:** Characteristics of SCM therapeutics.

	Sasang constitutional medicine (SCM)
	*Basis*
Basic application	Im et al. [53]: The outwardly visible symptoms due to external pathogen invasion are not the most important factor when treating. The most important factor in understanding that patient's zang and fu organs are congenital weaknesses and strengths Chae et al. [8]: Sasang typology offers temperament-based guidelines for the safe and effective use of herbal medicine, including even herbs with significant adverse effects such as ma huang (*Ephedra sinica*) [54] and aconite (*Aconitum carmichaelii*) [55]

	*Restore the Original Balance*
	Song [56]: Water-Food (nutrient) metabolism drawing in and sending out of nutrient material is related to the physiology, pathology, and therapeutics of Soyang and Soeum types (in the superior-inferior dimension). The Energy-Fluid metabolism, the exhaling and inhaling of energy and fluid, is related to the physiology, pathology, and therapeutics of Taeyang and Taeeum types (in the interior-exterior dimension)
Principles	Song [57]: Pathogenesis of disease has an emotional dimension that effects the mind effecting the body. To control the mind and to prevent the mind from declining into vices, one must nurture one's understanding of others and rectify oneself. Nurturing an “understanding of others and rectifying oneself” is having proper knowledge and actions through the rejection of the inattentive mind and rejection of indolent conduct. By developing wisdom, one is able to prevent external diseases of Seong in oneself: by rectifying oneself, one is able to prevent internal diseases of Jeong in relationships with others Shim et al. [34]: A disease occurs when the inherent imbalance between body and mind are triggered by a certain external condition. Under this view, the physical constitution of a patient is diagnosed in advance, and then an understanding of the inherent imbalance of the patient should be the next step to be undertaken for treatment. Food and medicine to complement these imbalances can be prescribed while the mental state of the patient is maintained or treated
	*Comprehensive Management of Constitution-Based Complications *
	Song [57]: Constitutional pathology is based upon diagnosing and treating each patient's symptoms according to his constitution type and from the perspective of that constitution. This is different from western diagnosis and treatment because instead of treating a specific disease, SCM is treating the presenting symptoms and underlying constitutional weaknesses

	*Control and Containment of the Seong-Jeong of Sorrow, Anger, Joy, and Pleasure*
Self-cultivation	Song [58]: Control and containment of one's mind and one's disease is one of the unique foundational medical philosophies of SCM Kim and Song [59]: In SCM preventative medicine, more focus is placed upon a healthy mind over a healthy body
	Chae et al. [8]: The principle of SCM treatment is based on the reinforcement of the hypoactive visceral groups. Thus, medicinal herbs of SCM are divided into four groups which correspond to the four constitutions. Under this rule, medicinal herbs and herbal remedies belonging to a certain constitution cannot be used for others: otherwise, this can result in no effect or an adverse effect Song et al. [17]: Resolution and overcoming personal or social conflict is largely dependent on the degree of self-cultivation that the afflicted individual accomplishes, and this self-cultivation is accomplished through interaction with others and rectifying the self. This process of self-cultivation is de facto regulation of the mind that results in control and containment of the Seong-Jeong of sorrow, anger, joy, and pleasure

	*Balancing the Healthy Qi*
Goal	Chae et al. [8]: Sasang typology also employs the same herbs and acupuncture points, but is different from conventional traditional Chinese medicine, since it encompasses stable biopsychological temperaments alongside with sociological facets emphasizing development of one's character
	Lee [26], and Lee et al. [27]: Wellness is when the lesser organ's function is optimal. The physiological signs for determining optimal function of the weaker lesser organ are when sweat, digestion, urination and bowel movements are normal

Innovation beyond TCM therapeutics	*Taeeum Person and Taeyang Person Pathology and Therapeutics*
	Choi [19]: Zhang Zhongjing had barely grasped the concept of the Taeeum person pathology and therapeutics, while the Song, Yuan, and Ming physicians had revealed them partially: Zhu Zhenheng (1281–1358) had barely grasped the concept of the Taeyang person pathology and therapeutics and miscellaneous herbology texts carry only fragments of them
	Song et al. [17]: When extrapolating the six meridian diseases narrated by Zhang to the SCM pathology, the great yin meridian pattern, lesser yin meridian pattern, and reverting yin meridian pattern in TCM most closely describes the Soeum person disease pattern in SCM. The lesser yang meridian pattern in TCM most closely describe the Soyang person disease pattern in SCM. The great yang meridian pattern and Yang brightness meridian pattern most closely describe the Soyang person, Soeum person, and Taeeum person disease patterns, especially the Soeum person disease pattern. It can be seen here that Zhang's theory of the six meridian diseases includes most of the Soeum person and Soyang person disease patterns, which are based on the Water-Food metabolism, but lacks explanations for most of the Taeeum person and the Taeyang person disease patterns, which are based on the Qi-Humor metabolism *Constitutional Approach of SCM and Tailored Medicine * Kim et al. [12], Kim and Pham [13], and Kim et al. [14]: SCM shares the same vision with tailored medicine—that individuals are not only can be cared for with individualized therapy, which takes into account entirely their distinctive factors, but individuals also can prevent specific susceptible chronic diseases and live healthily by personalized self-regulation. The shortcomings of an individual due to the hypoactive visceral group should be controlled by specific constitutional medication, diet, physical training and psychological caution *Pharmacology * Leem and Park [18]: Initially pharmacological theory focused on using *materia medica* such as the four qi's and five tastes for supporting the associated lesser hypoactive organ. Later, this theory was fine tuned and expanded into a pharmacological theory which stated that materia medica should be chosen specifically according to the constitution of the patient support not only the lesser hypoactive organ but also the greater hyperactive organ as well. Pharmacological scope was broadened to treat both constitutionally significant organs Seo et al. [60]: Pharmacological theory focused on the functional health of the organs over the pharmacological qualities and characteristics of the herbs. This was because Lee Jema emphasized the conservative use of herbs. Lee Jema viewed that herbs were secondary to the body's own ability to recover. It was highly important to be able to evaluate the state of the organ. Other characteristics used were identifying whether the disease was recent or chronic and whether the disease was severe or mild
